# RIPK1 inhibition attenuates experimental autoimmune arthritis via suppression of osteoclastogenesis

**DOI:** 10.1186/s12967-019-1809-3

**Published:** 2019-03-15

**Authors:** Jooyeon Jhun, Seung Hoon Lee, Se-Young Kim, Jaeyoon Ryu, Ji Ye Kwon, Hyun Sik Na, KyoungAh Jung, Su-Jin Moon, Mi-La Cho, Jun-Ki Min

**Affiliations:** 1 The Rheumatism Research Center, Catholic Research Institute of Medical Science, The Catholic University of Korea, Seoul, South Korea; 2 Department of Immunology, Blavatnik Institute, Harvard Medical School, Boston, MA USA; 3 Impact Biotech, Seoul, 137-040 South Korea; 4 Laboratory of Immune Network, Conversant Research Consortium in Immunologic Disease, College of Medicine, The Catholic University of Korea, Seoul, South Korea; 5 Department of Internal Medicine, The Clinical Medicine Research Institute of Bucheon St. Mary’s Hospital, Bucheon-si, South Korea

**Keywords:** Rheumatoid arthritis, Necrostatin-1s, Osteoclastogenesis, Necroptosis

## Abstract

**Background:**

Rheumatoid arthritis (RA) is a chronic and systemic inflammatory disease characterized by upregulation of inflammatory cell death and osteoclastogenesis. Necrostatin (NST)-1s is a chemical inhibitor of receptor-interacting serine/threonine-protein kinase (RIPK)1, which plays a role in necroptosis.

**Methods:**

We investigated whether NST-1s decreases inflammatory cell death and inflammatory responses in a mouse model of collagen-induced arthritis (CIA).

**Results:**

NST-1s decreased the progression of CIA and the synovial expression of proinflammatory cytokines. Moreover, NST-1s treatment decreased the expression of necroptosis mediators such as RIPK1, RIPK3, and mixed lineage kinase domain-like (MLKL). In addition, NST-1s decreased osteoclastogenesis in vitro and in vivo. NST-1s downregulated T helper (Th)1 and Th17 cell expression, but promoted Th2 and regulatory T (Treg) cell expression in CIA mice.

**Conclusions:**

These results suggest that NST-1s attenuates CIA progression via the inhibition of osteoclastogenesis and might be a potential therapeutic agent for RA therapy.

## Background

Rheumatoid arthritis (RA) is a progressive autoimmune polyarthritis disease characterized by severe bone loss and inflammatory cell infiltration in the affected joints. The excessive production of proinflammatory cytokines is involved in the pathogenesis of RA. Clinically, interleukin (IL)-17 and tumor necrosis factor (TNF)-α are increased in the serum and arthritic synovium in patients with RA [1, 2].

Osteoclasts are large multinucleated cells; these bone-resorbing macrophage lineage cells polykaryons play an important role in bone destruction [3]. The etiology of RA is complex and the mechanisms involved are unclear; however, osteoclastogenesis is a significant target for RA treatment, as osteoclasts cause destruction of bone and cartilage. Indeed, osteoclastogenesis is increased in patients with RA compared to healthy individuals [4]. In addition, proinflammatory cytokines, such as IL-17 and TNF-α, increase osteoclastogenesis [5, 6]. T helper (Th)17 cells that release IL-17 mainly aggravate osteoclastogenesis for bone resorption [7].

Necroptosis, a type of cell death that is a programmed form of necrosis, induces pronounced inflammation [8]. Receptor-interacting serine/threonine-protein kinase (RIPK)1 is a member of the TNF receptor-I signaling complex that causes necroptosis [9]. Inflammatory cell death results in the pathophysiological conditions seen in several inflammatory diseases [10, 11]. In addition, necroptosis is increased in the synovium of animals with experimental autoimmune arthritis [12].

Necrostatin (NST)-1s, a chemical inhibitor for RIPK1 has been recognized as a potent necroptosis inhibitor. There are several evidences that inhibition of RIPK1 can improve inflammatory response and decrease necroptotic cell death in vitro and in vivo [13, 14]. Recently, NST-1s reduces inflammatory response and enhances tissue repair in aortic aneurysm model [15].

We investigated whether the inhibition of RIPK1 ameliorates osteoclastogenesis and inflammatory responses in an experimental autoimmune arthritis mouse model with collagen-induced arthritis (CIA). Furthermore, we investigated whether RIPK1 inhibitor decreases CIA by suppressing osteoclastogenesis and joint inflammation. We examined the expression of osteoclasts in vitro and in vivo. In addition, we analyzed the protective activity of RIPK1 inhibitor against joint inflammation and measured the production of necroptosis markers in CIA mice.

## Methods

### Mice

Seven-week-old male DBA/1J mice (Orient Bio, Gyeonggi-do, Korea) were maintained under specific pathogen-free conditions and fed standard laboratory mouse chow (Ralston Purina, St. Louis, MO, USA) and water ad libitum. The animals were housed five per cage in a room maintained under controlled temperature (21–22 °C) and lighting (12 h light/dark cycle) conditions. All experimental procedures were approved by the Institutional Animal Care and Use Committee at the School of Medicine, Animal Research Ethics Committee of the Catholic University of Korea and were conducted in accordance with the Laboratory Animals Welfare Act according to the Guide for the Care and Use of Laboratory Animals.

### Induction of arthritis and treatment

CIA was induced in DBA1/J mice (n = 5). The experiment was performed three times. Type II collagen (CII) was dissolved overnight in 0.1 N acetic acid (4 mg/mL) with gentle rotation at 4 °C. Male DBA/1J mice were immunized intra-dermally at the base of the tail with 100 mg of chicken CII (Chondrex, Inc., Remosa, WA, USA) in complete Freund’s adjuvant (Chondrex Inc.). In experiments conducted to investigate preventive effects, mice were boosted with 100 mg of CII emulsified with incomplete Freund’s adjuvant (Chondrex Inc.), injected intradermally into the base of the tail on day 14 after primary immunization. The arthritis model mice were injected intraperitoneally (i.p.) with necrostatin (NST)-1s (Merck Millipore) or saline control (vehicle) starting on day 4 after the first immunization. The mice were examined visually twice weekly for the appearance of arthritis in the peripheral joints. Mice were sacrificed on week 14 for histological analyses of splenocytes and determination of protein expression.

### Clinical assessment of arthritis

The severity of arthritis was recorded using the mean arthritis index on a scale of 0–4, as follows: (0), no evidence of erythema and swelling [1]; erythema and mild swelling confined to the midfoot (tarsals) or ankle joint [2]; erythema and mild swelling extending from the ankle to the midfoot [3]; erythema and moderate swelling extending from the ankle to the metatarsal joints; and [4], erythema and severe swelling encompassing the ankle, foot and digits. The severity of arthritis was given by the sum of the scores from all legs, as assessed by two independent observers with no knowledge of the experimental groups.

### Histological analyses

Mouse joint tissues were fixed in 4% paraformaldehyde (Sigma-Aldrich, St. Louis, MO, USA), decalcified in histological decalcifying agent (Calci-Clear Rapid; National Diagnostics, Atlanta, GA, USA), trimmed, and embedded in paraffin wax. Sections (7 μm) were prepared and stained with hematoxylin (YD Diagnostics, Yongin, Korea), eosin (Muto Pure Chemicals Co., Ltd., Tokyo, Japan), and Safranin O (Sigma-Aldrich). Cartilage damage was scored as described previously [16].

### Immunohistopathological analyses of arthritis

Joint tissues were first incubated with primary antibodies against IL-1β (R&D Systems, Minneapolis, MN, USA), IL-6 (R&D Systems), TNF-α (R&D Systems), IL-17 (R&D Systems), RIPK1 (Cell Signaling Technology, Danvers, MA, USA), RIPK3 (Cell Signaling), pMLKL (Cell Signaling), tartrate-resistant acid phosphatase (TRAP; R&D Systems), receptor activator of nuclear factor kappa-B (RANK; R&D Systems) and RANK ligand (RANKL; R&D Systems) overnight at 4 °C. Samples were incubated with a biotinylated secondary antibody, followed by incubation with a streptavidin–peroxidase complex for 1 h. Samples were then developed using chromogen 3,3′-diaminobenzidine (Thermo Scientific, Rockford, IL, USA). The sections were examined under a photomicroscope (Olympus, Tokyo, Japan). The number of positive cells was counted using Adobe Photoshop software (Adobe, USA) on high-power digital image (magnification: 400×). Positive cells were enumerated visually by three individuals, and the mean values were calculated.

### Confocal microscopy of immunostaining

Spleen tissues were obtained at 14 weeks after the first immunization. Tissues were snap-frozen in liquid nitrogen and stored at − 80 °C. Tissue sections were fixed in 4% paraformaldehyde and stained with anti-CD4, FITC-conjugated anti-forkhead box P3 (Foxp3), APC-conjugated anti-CD25, PE-conjugated anti-IL-17, PE-conjugated anti-interferon (IFN)-r and PE-conjugated anti-IL-4 (all from eBiosciences, San Diego, CA, USA). Stained sections were analyzed using a Zeiss microscope (LSM510Meta; Carl Zeiss, Oberkochen, Germany).

### Flow cytometry analysis

Expression of cytokines in mice was assessed by intracellular staining with the following antibodies: fluorescein isothiocyanate (FITC)-conjugated anti-IL-17, phycoerythrin (PE)-conjugated anti-Foxp3, Percp-conjugated anti-CD4, allophycocyanin (APC)-conjugated anti-CD25, APC-conjugated anti-IFN-r, and PE-conjugated anti-IL-4. Cells were stimulated for 4 h with phorbol myristate and ionomycin, with the addition of GolgiStop (BD Bioscience, San Diego, CA, USA). The cultured cells were surface-labeled for 30 min and then permeabilized with Cytofix/Cytoperm solution (BD Bioscience). Thereafter, the cells were intracellularly stained with fluorescent antibodies before FACS Calibur flow cytometry analyses. The data were collected and analyzed with FlowJo software (Tree Star, Ashland, OR, USA).

### In vitro osteoclastogenesis

Bone marrow cells from mouse femurs were cultured in alpha-minimal essential medium (Invitrogen, Burlingame, CA, USA) containing antibiotics and 10% heat-inactivated fetal bovine serum to separate the floating and adherent cells. Nonadherent cells were washed away, and preosteoclasts were further cultured in the presence of 10 ng/mL macrophage colony-stimulating factor (M-CSF), 100 ng/mL receptor activator of RANKL (PeproTech, London, UK) and NST-1s for 4 days to generate osteoclasts. The medium was changed every 2 d. Osteoclasts were generated after 8–10 days.

### Cell viability assay

The cell cytotoxicity was assessed using Cell Counting Kit-8 (CCK-8, Dojindo Molecular Technologies, Inc.). Cells (2 × 10^5^) treated 20 μl CCK-8 solution per well for the final 4 h of the 72-h culture period. Optical density measured at 450 nm.

### TRAP staining

A commercial TRAP kit (Sigma-Aldrich) was used according to the manufacturer’s instructions; however, counterstaining with hematoxylin was not performed. TRAP-positive multinuclear cells (MNCs) containing three or more nuclei were counted as osteoclasts.

### Gene expression analyses using real-time polymerase chain reaction (PCR)

PCR amplification and analyses were performed using a LightCycler 2.0 instrument (Roche Diagnostics, Mannheim, Germany) with software version 4.0. All reactions were performed using LightCycler Fast Start DNA Master SYBR green I (Takara, Shiga, Japan), according to the manufacturer’s instructions. The following primers were used: TRAP, 5′-TCC TGG CTC AAA AAG CAG TT-3′ (sense) and 5′-ACA TAG CCC ACA CCG TTC TC-3′ (antisense); calcitonin receptor, 5′-CGG ACT TTG ACA CAG AA-3′ (sense) and 5′-AGC AAT CGA CAA GGA GT-3′ (antisense); integrin b3, 5′-CTG TGG GCT TTA AGG ACA GC-3′ (sense) and 5′-GAG GGT CGG TAA TCC TC-3′ (antisense); cathepsin K, 5′-CAG AGG TGT GTA CTA TG-3′ (sense) and 5′-GCG TTG TTC TTA TTC CGA GC-3′ (antisense); RIPK1, 5′-CTG TTC CCT GTG CCC AAT AA-3′ (sense) and 5′-ATG ACT CTG AAG CTG TCC TTT C-3′ (antisense) and RIPK3, 5′-GCA CTC CTC AGA TTC CAC ATA C-3′ (sense) and 5′-GTG TCT TCC ATC TCC CTG ATT C-3′ (antisense).

### Statistical analyses

Statistical analyses were conducted using the nonparametric Mann–Whitney *U* test for comparisons between two groups, and one-way ANOVA with Bonferroni’s post hoc test for multiple comparisons. GraphPad Prism (ver. 5.01; GraphPad Software Inc., San Diego, CA, USA) was used for all analyses. p < 0.05 was used as the threshold for statistical significance. The data are presented as mean ± standard deviation (SD).

## Results

### NST-1s shows therapeutic activity in CIA mice

**Fig. 1:**
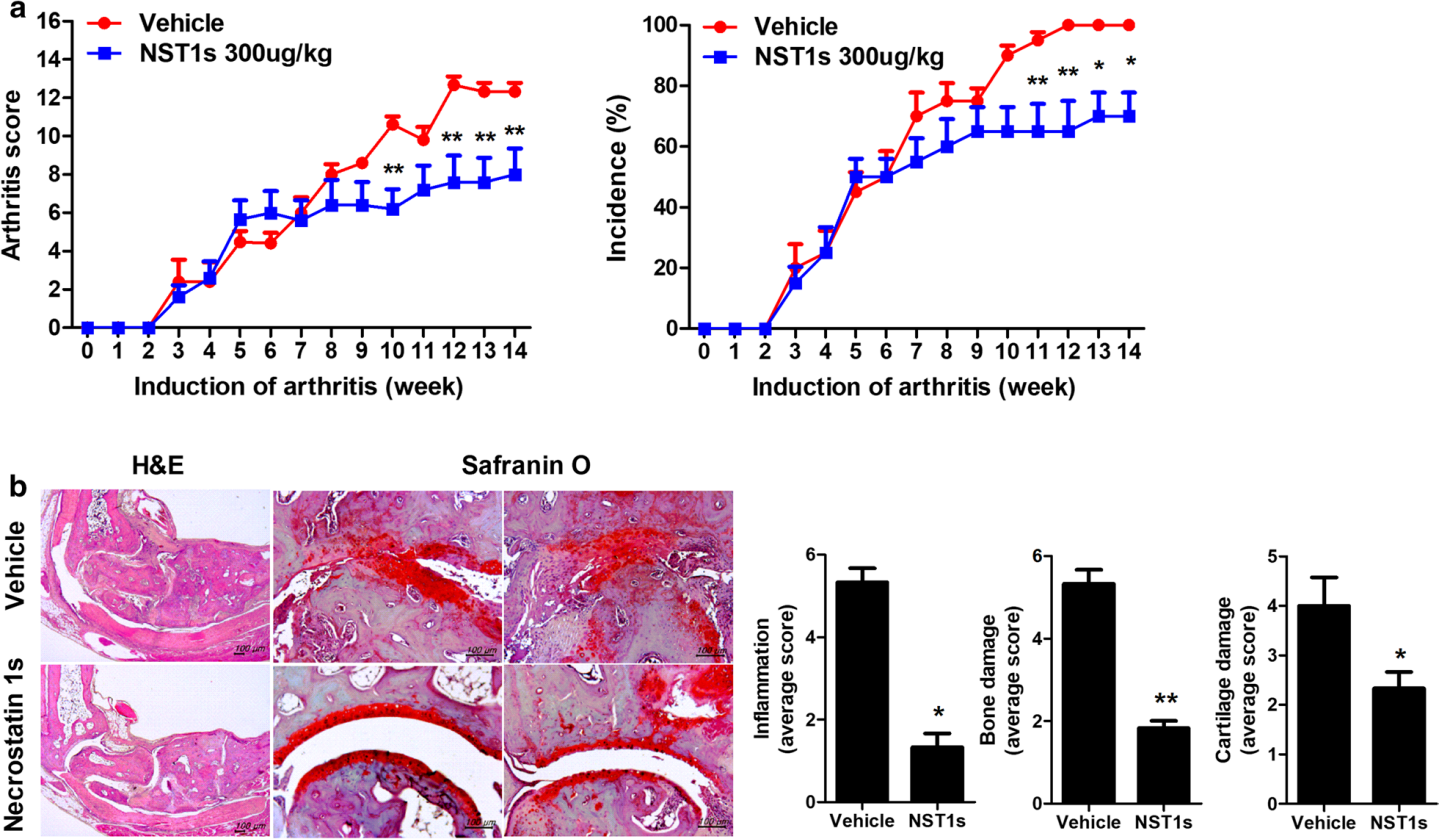
Necrostatin-1s (NST-1s) inhibits development of rheumatoid arthritis (RA) in the collagen-induced arthritis (CIA) mouse model. **a** Reduction in arthritis score and arthritis incidence in CIA mice treated with NST-1s. CIA mice were injected intraperitoneally (i.p.) with NST-1s (300 µg/kg) once daily for 14 weeks after CIA induction. **b** Representative results of the effects of NST-1s on RA development in CIA mice. Tissue specimens obtained from the hind paw joints of each group of mice at the end point of the experiment were analyzed by staining with hematoxylin and eosin (H&E) and Safranin O. *p < 0.05; **p < 0.01; ***p < 0.001

**Fig. 2:**
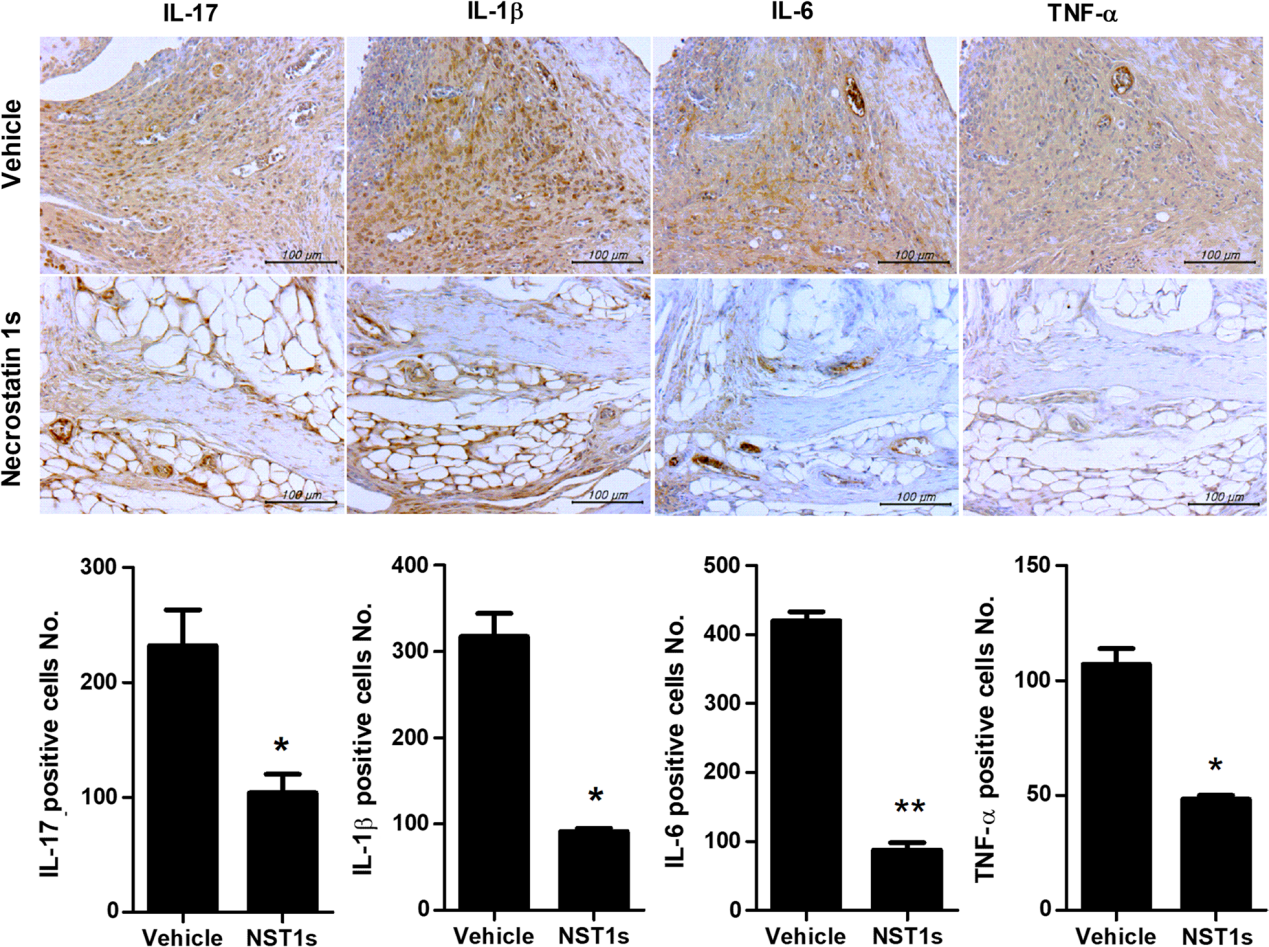
NST-1s decreases inflammatory cytokine expression, including interleukin (IL)-17, IL-1b, IL-6 and tumor necrosis factor (TNF)-α. The IHC results showed that NST-1s reduced collagen induced arthritis compared to the vehicle control. Inflammatory factors were downregulated. Immunohistochemical staining was used to detect IL-6, IL-17, IL-1β and TNF-α in the synovium of CIA mice (vehicle or NST-1s; scale bar, 100 μM). *p < 0.05; **p < 0.01; ***p < 0.001

Mice were injected i.p. with either NST-1s or DMSO as a vehicle at 1 week after immunization with CII (Fig. 1a). Administration of NST-1s decreased the arthritis score and showed a protective function in the arthritic tissues of the affected joints (Fig. 1b, c). Proinflammatory cytokines, such as IL-17, IL-1β, IL-6 and TNF-α in the arthritic joint were decreased by NST-1s treatment (Fig. 2a). Moreover, infiltration of CD4+ T cells in vehicle group joint is much higher than that of NST-1s group joint because NST-1s improved CIA, thus CD4+ T cells infiltration was also decreased in NST-1s group joint (data not shown). These results suggest that NST-1s diminished the development of CIA by inhibiting joint damage and the expression of inflammatory cytokines and mediators of necroptosis.

### NST-1s suppresses the expression of necroptosis factors in CIA mice

**Fig. 3:**
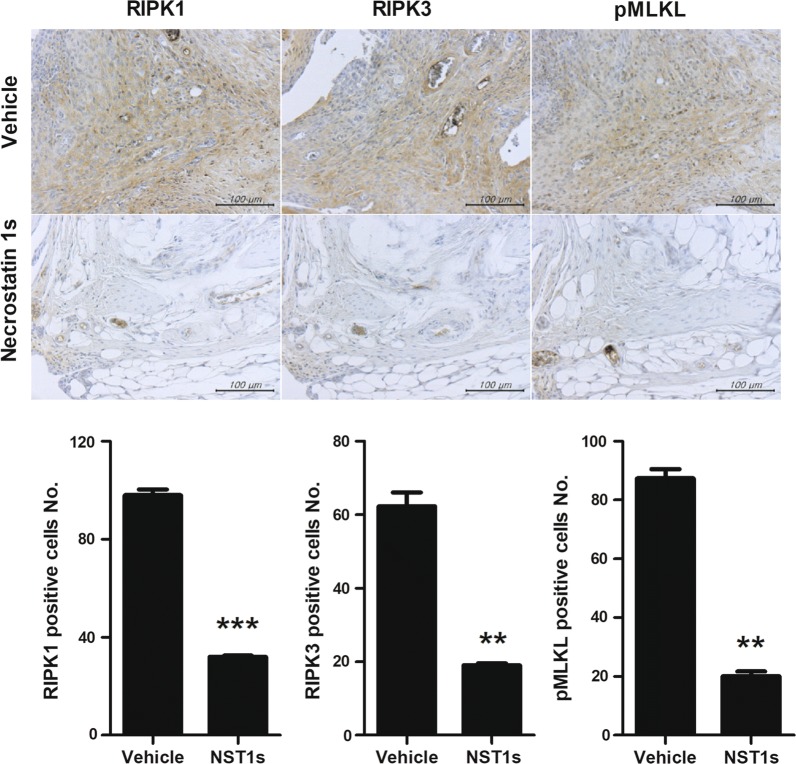
NST-1s suppresses necrotic cell death by decreasing the expression of receptor-interacting serine/threonine-protein kinase (RIPK)1, RIPK3, and mixed lineage kinase domain-like (pMLKL). Synovial tissue from CIA-induced control and NST-1s treated mice were subjected to immunohistochemical staining RIPK1, RIPK3, and pMLKL. The cells showing positively for RIPK1, RIPK3, and pMLKL were visually presented at a higher magnification. *p < 0.05; **p < 0.01; ***p < 0.001

NST-1s downregulated the expression of necroptosis mediators such as RIPK1, 3 and pMLKL in the synovium of CIA mice (Fig. 3a). To investigate whether NST-1s inhibits necroptosis, we performed in vitro experiments in mice joints.

### NST-1s decreases inflammatory T cells, but increases anti-inflammatory T cells, in CIA mice

**Fig. 4:**
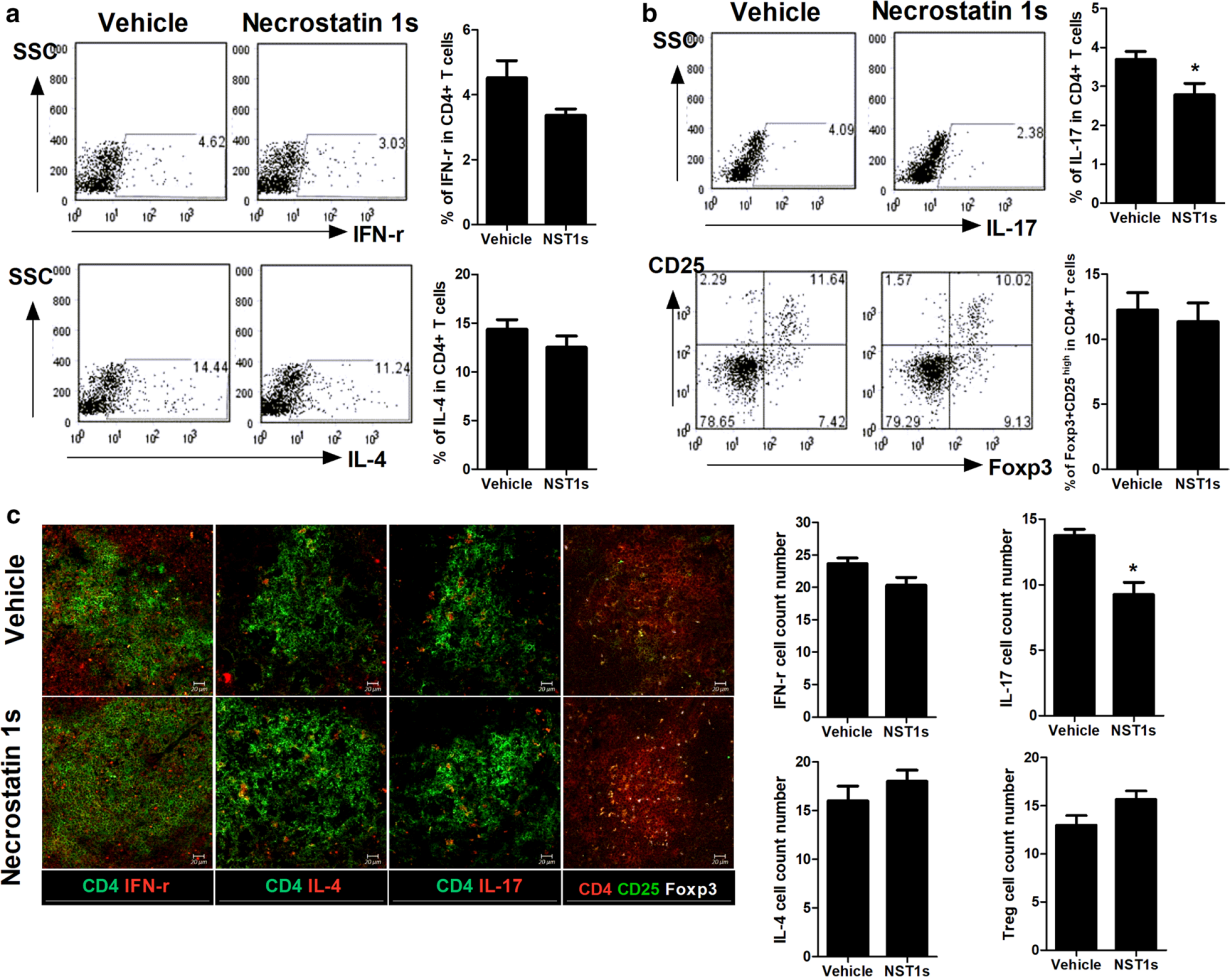
NST-1s represses IL-17 but maintains Foxp3 expression in CD4 + T cells in CIA mice. **a**, **b** Flow cytometric analysis shows the Th1 cells (CD4+ IFN-r+), Th2 cells (CD4+ IL-4+), Th17 cells (CD4+ IL17+) and Tregs (CD4+ CD25+ FOXP3+) in splenocytes of CIA mice. **c** Spleen tissues from each mice were stained for Th1, Th2, Th17 and Tregs were enumerated visually at higher magnification, and the mean values are presented in the form of histogram (right panel). *p < 0.05; **p < 0.01; ***p < 0.001

NST-1s decreased the expression of IFN-γ and IL-17, but not IL-4 and Foxp3 in CD4^+^ T cells from mice splenocytes (Fig. 4a, b). In addition, NST-1s decreased the population of Th17 cells, but increased that of Treg cells (Fig. 4c). But, there was no big difference of the numbers of CD4+ T cells in spleens between vehicle and NST-1s. These results demonstrated that NST-1s attenuates the inflammatory response in CIA mice through the reciprocal balance between inflammatory T cells and anti-inflammatory T cells.

### NST-1s reduces osteoclastogenesis in vitro and in CIA mice

**Fig. 5:**
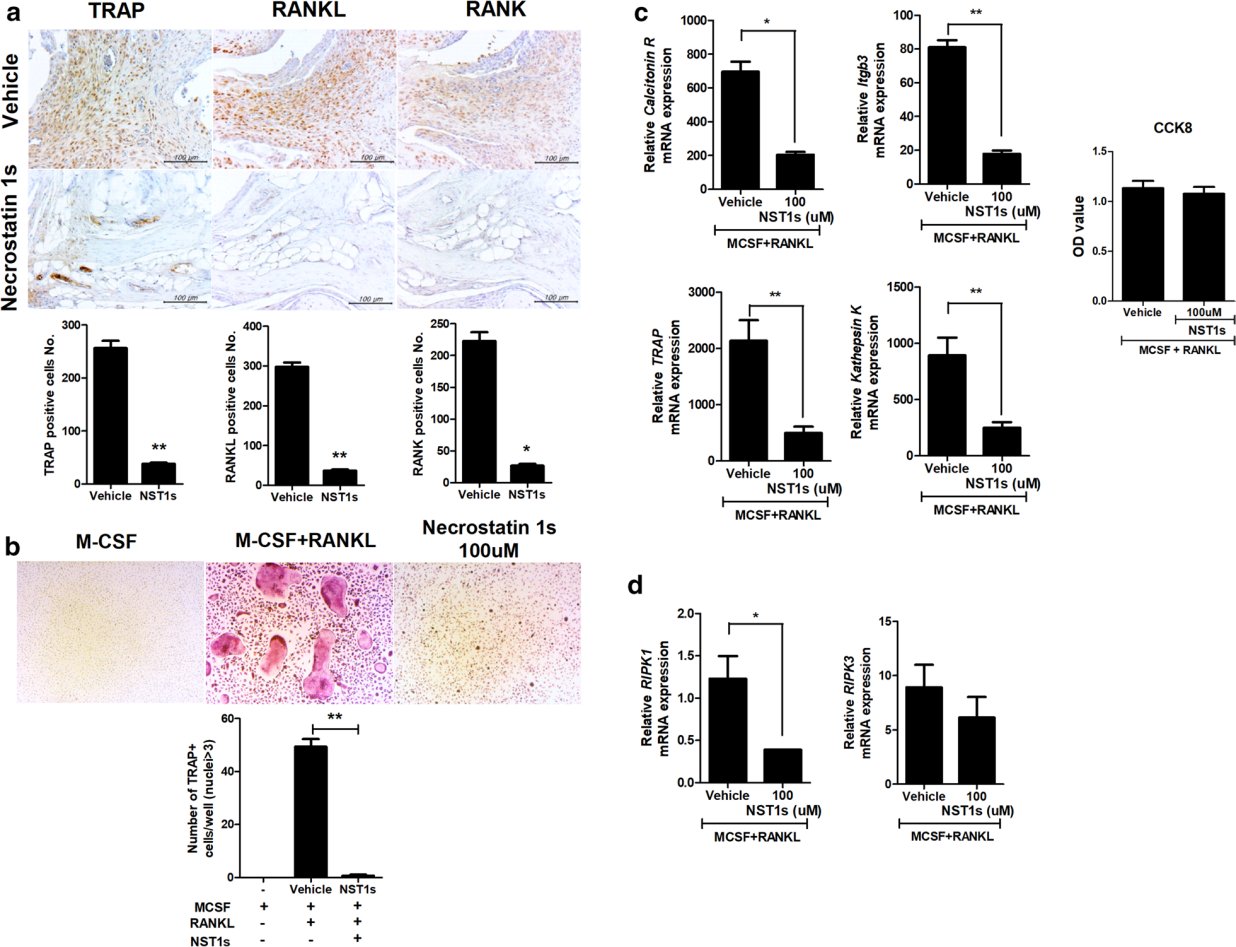
NST-1s attenuates CIA through the suppression of osteoclastogenesis. **a** Suppression of cytokines involved in the destruction of joints in NST-1s-treated mice with autoimmune arthritis ankle joints. Tissues from CIA mice and NST-1s-injected mice were obtained at 14 weeks and stained immunohistochemically with specific antibodies to tartrate-resistant acid phosphatase (TRAP), receptor activator of nuclear factor kappa-B (RANK) and RANK ligand (RANKL). **b** Murine monocytes obtained from the femur and tibia were cultured with macrophage colony-stimulating factor (M-CSF) and RANKL to induce osteoclastogenesis. TRAP-positive multinucleated cells were counted in the culture dishes with or without 100 µM of NST-1s. **c**, **d** The relative mRNA levels of osteoclastogenesis-related markers (TRAP, integrin beta chain beta 3, cathepsin K and calcitonin receptor), RIPK1 and RIPK3 were evaluated using reverse transcription polymerase chain reaction. Osteoclasts were cultured with NST-1s 100 μM for 72 h and cell viability was analyzed by CCK-8 analysis. *p < 0.05; **p < 0.01; ***p < 0.001

Osteoclastogenesis is measured by TRAP staining because TRAP is a marker for osteoclasts [17]. It has been suggested that TRAP-positive MNCs containing three or more nuclei are known as osteoclasts [18, 19]. NST-1s reduced the number of TRAP, RANK and RANKL positive cells in CIA mice (Fig. 5a). Mouse bone-marrow cells were stimulated with M-CSF and RANKL to induce osteoclastogenesis with without NST-1s; NST-1s inhibited the formation of osteoclastogenesis in vitro (Fig. 5b). The relative mRNA levels of osteoclastogenesis markers were significantly decreased by NST-1s in vitro (Fig. 5c). Osteoclast cell viability was analyzed after an 72 h exposure to NST-1 100 μM. A cytotoxicity assay indicated that NST-1 did not affect cell viability (Fig. 5c). Thus, NST-1s diminished CIA by inhibiting osteoclastogenesis. Treatment with NST-1s significantly reduced the level of RIPK1 gene. However, The level of RIPK3 gene expression was not difference (Fig. 5d).

## Discussion

Many studies have investigated the suppressive role of RIPK1 inhibitor in cell death [20]. RIPK1 inhibitor improves intestinal inflammatory responses and decreases T cell receptor activation [14, 21]. However, no studies have investigated whether RIPK1 inhibitor functions in the T cell-mediated inflammatory responses and autoimmune arthritis involved in osteoclastogenesis. Furthermore, little is known about the therapeutic activity of NST-1s in inflammatory autoimmune diseases. In this study, we examined the therapeutic function of NST-1s in CIA. In addition, we investigated the mechanistic role of NST-1s in osteoclastogenesis in vitro and in vivo.

NST-1s diminished CIA through the suppression of osteoclastogenesis. RA is characterized by osteoclastogenesis in the synovium, which is associated with erosive polyarthritis [3]. Previous reports revealed that the inhibition of osteoclastogenesis attenuates CIA progression [18, 19, 22]. Our results suggest that NST-1s suppressed osteoclastogenesis, which is known to attenuate RA development. This finding indicates that NST-1s may be a potential therapeutic agent to treat RA via RIPK1 inhibition.

Necroptosis is a significant factor involved in CIA development. Indeed, the expression of RIPK1, RIPK3, and MLKL was increased in the synovium in a CIA model [12]. As necroptosis can induce a massive inflammatory response and RIPK1 regulates inflammatory cell death [8, 23], NST-1s might decrease necroptosis mediators in the synovium of a CIA model. In this study, NST-1s decreased the production of proinflammatory cytokines in the synovium of CIA mice. These results suggest that RIPK1 inhibition can attenuate the inflammatory response in CIA through inhibition of necroptosis.

Osteoclastogenesis is related with the activity of nuclear factor-kappa B (NF-κB). It is well reported that NF-κB activation is required for osteoclast formation [24]. RIPK1 is also involved in nuclear translocation of NF-κB [25]. Th17 cells can stimulate osteoclast precursors and cause osteoclastogenesis [26]. It has been suggested that the inhibition of Th17 cells differentiation reduced TRAP expression in joint from CIA mice [18]. Recently, NST-1 in low dose such as 0.1 and 1 μM didn’t decrease osteoclastogenesis in vitro [27]. Also, we observed no significant effects of 10uM NST-1s. But, we found the inhibitory effect of NST-1s for osteoclastogenesis in high dose such as 100 μM. In addition, inhibitory activity for p-RIPK1 of NST-1s is more effective than that of NST-1 [28]. We also observed that NST-1s decreases TRAP expression and Th17 cells frequency in vivo. These observations demonstrate that RIPK1 suppression can improve CIA through inhibition of osteoclastogenesis.

Th17 cells are pathogenic factors in several autoimmune diseases, and inhibition of Th17 cell differentiation diminishes inflammatory disorders [29–31]. Downregulation of Th17 cell populations leads to attenuation of CIA [32]. On the other hand, Treg cells show an immunosuppressive function; thus, a reciprocal balance between Th17 and Treg cells is important to diminishing CIA [18, 19]. Moreover, suppression of Th17 cell frequency decreased osteoclasts in a CIA model [33]. In our study, NST-1s reduced Th17 cell differentiation and CIA progression.

## Conclusion

To the best of our knowledge, the present study is the first to demonstrate the potential for NST-1s as a therapeutic agent in autoimmune arthritis, as CIA was diminished by NST-1s treatment. NST-1s showed therapeutic activity by suppressing osteoclastogenesis and the expression of proinflammatory cytokines and mediators of necroptosis. In addition, this study showed that RIPK1 has an important role in RA pathogenesis. Therefore, RIPK1 inhibition might be a potential therapeutic target for RA therapy.

## Abbreviations

CIA: collagen induced arthritis
RA: rheumatoid arthritis
RIPK1: receptor-interacting serine/threonine-protein kinase 1
MLKL: mixed lineage kinase domain-like C
IL: interleukine
Treg: T regulatory
TRAP: tartrate-resistant acid phosphatase
RANK: receptor activator of nuclear factor kappa-B
RANKL: RANK ligand
M-CSF: macrophage colony-stimulating factor

## References

[1] Ziolkowska M, Koc A, Luszczykiewicz G, Ksiezopolska-Pietrzak K, Klimczak E, Chwalinska-Sadowska H (2000). High levels of IL-17 in rheumatoid arthritis patients: IL-15 triggers in vitro IL-17 production via cyclosporin A-sensitive mechanism. J Immunol..

[2] Brennan FM, McInnes IB (2008). Evidence that cytokines play a role in rheumatoid arthritis. J Clin Invest..

[3] Schett G (2007). Cells of the synovium in rheumatoid arthritis, osteoclasts. Arthritis Res Ther..

[4] Durand M, Boire G, Komarova SV, Dixon SJ, Sims SM, Harrison RE (2011). The increased in vitro osteoclastogenesis in patients with rheumatoid arthritis is due to increased percentage of precursors and decreased apoptosis—the in vitro osteoclast differentiation in arthritis (IODA) study. Bone.

[5] Yago T, Nanke Y, Ichikawa N, Kobashigawa T, Mogi M, Kamatani N (2009). IL-17 induces osteoclastogenesis from human monocytes alone in the absence of osteoblasts, which is potently inhibited by anti-TNF-alpha antibody: a novel mechanism of osteoclastogenesis by IL-17. J Cell Biochem.

[6] Lam J, Takeshita S, Barker JE, Kanagawa O, Ross FP, Teitelbaum SL (2000). TNF-alpha induces osteoclastogenesis by direct stimulation of macrophages exposed to permissive levels of RANK ligand. J Clin Invest..

[7] Sato K, Suematsu A, Okamoto K, Yamaguchi A, Morishita Y, Kadono Y (2006). Th17 functions as an osteoclastogenic helper T cell subset that links T cell activation and bone destruction. J Exp Med.

[8] Pasparakis M, Vandenabeele P (2015). Necroptosis and its role in inflammation. Nature.

[9] Silke J, Rickard JA, Gerlic M (2015). The diverse role of RIP kinases in necroptosis and inflammation. Nat Immunol.

[10] Linkermann A, Green DR (2014). Necroptosis. N Engl J Med.

[11] Vanden Berghe T, Linkermann A, Jouan-Lanhouet S, Walczak H, Vandenabeele P (2014). Regulated necrosis: the expanding network of non-apoptotic cell death pathways. Nat Rev Mol Cell Biol.

[12] Lee SH, Kwon JY, Kim SY, Jung K, Cho ML (2017). Interferon-gamma regulates inflammatory cell death by targeting necroptosis in experimental autoimmune arthritis. Sci Rep..

[13] Zhang S, Wang Y, Li D, Wu J, Si W, Wu Y (2016). Necrostatin-1 attenuates inflammatory response and improves cognitive function in chronic ischemic stroke mice. Medicines (Basel)..

[14] Liu ZY, Wu B, Guo YS, Zhou YH, Fu ZG, Xu BQ (2015). Necrostatin-1 reduces intestinal inflammation and colitis-associated tumorigenesis in mice. Am J Cancer Res..

[15] Wang Q, Zhou T, Liu Z, Ren J, Phan N, Gupta K (2017). Inhibition of Receptor-Interacting Protein Kinase 1 with Necrostatin-1s ameliorates disease progression in elastase-induced mouse abdominal aortic aneurysm model. Sci Rep..

[16] Camps M, Ruckle T, Ji H, Ardissone V, Rintelen F, Shaw J (2005). Blockade of PI3Kgamma suppresses joint inflammation and damage in mouse models of rheumatoid arthritis. Nat Med.

[17] Hayman AR (2008). Tartrate-resistant acid phosphatase (TRAP) and the osteoclast/immune cell dichotomy. Autoimmunity..

[18] Lee SH, Kim EK, Kwon JE, Lee JK, Lee D, Kim SY (2017). Ssu72 attenuates autoimmune arthritis via targeting of STAT3 signaling and Th17 activation. Sci Rep..

[19] Lee SH, Park JS, Byun JK, Jhun J, Jung K, Seo HB (2016). PTEN ameliorates autoimmune arthritis through down-regulating STAT3 activation with reciprocal balance of Th17 and Tregs. Sci Rep..

[20] Degterev A, Huang Z, Boyce M, Li Y, Jagtap P, Mizushima N (2005). Chemical inhibitor of nonapoptotic cell death with therapeutic potential for ischemic brain injury. Nat Chem Biol.

[21] Cho Y, McQuade T, Zhang H, Zhang J, Chan FK (2011). RIP1-dependent and independent effects of necrostatin-1 in necrosis and T cell activation. PLoS ONE.

[22] Lee SY, Lee SH, Park SJ, Kim DJ, Kim EK, Kim JK (2016). (p40)2-Fc reduces immune-inflammatory response through the activation of T cells in collagen induced arthritis mice. Immunol Lett.

[23] Newton K (2015). RIPK1 and RIPK3: critical regulators of inflammation and cell death. Trends Cell Biol.

[24] Boyce BF, Xiu Y, Li J, Xing L, Yao Z (2015). NF-kappaB-mediated regulation of osteoclastogenesis. Endocrinol Metab (Seoul)..

[25] Kondylis V (2017). RIPK1 and allies in the battle against hepatocyte apoptosis and liver cancer. Transl Cancer Res.

[26] Adamopoulos IE, Bowman EP (2008). Immune regulation of bone loss by Th17 cells. Arthritis Res Ther..

[27] Fuji H, Ohmae S, Noma N, Takeiri M, Yasutomi H, Izumi K (2018). Necrostatin-7 suppresses RANK-NFATc1 signaling and attenuates macrophage to osteoclast differentiation. Biochem Biophys Res Commun.

[28] Takahashi N, Duprez L, Grootjans S, Cauwels A, Nerinckx W, DuHadaway JB (2012). Necrostatin-1 analogues: critical issues on the specificity, activity and in vivo use in experimental disease models. Cell Death Dis..

[29] Park MJ, Lee SH, Lee SH, Kim EK, Lee EJ, Moon YM (2016). GRIM19 ameliorates acute graft-versus-host disease (GVHD) by modulating Th17 and Treg cell balance through down-regulation of STAT3 and NF-AT activation. J Transl Med..

[30] Lee SH, Park MJ, Lee SH, Cho ML (2016). Coenzyme Q10 exerts anti-inflammatory activity and induces treg in graft versus host disease. J Med Food.

[31] Lee SY, Lee SH, Yang EJ, Kim JK, Kim EK, Jung K (2017). Coenzyme Q10 inhibits Th17 and STAT3 signaling pathways to ameliorate colitis in mice. j med food.

[32] Lee SH, Moon YM, Seo HB, Kim SY, Kim EK, Yi J (2016). HtrA2 suppresses autoimmune arthritis and regulates activation of STAT3. Sci Rep.

[33] Jhun J, Lee SH, Byun JK, Jeong JH, Kim EK, Lee J (2015). Coenzyme Q10 suppresses Th17 cells and osteoclast differentiation and ameliorates experimental autoimmune arthritis mice. Immunol Lett.

